# Development of an In Situ Analyzer Based on Sequential Injection Analysis and Liquid Waveguide Capillary Flow Cell for the Determination of Dissolved Reactive Phosphorus in Natural Waters

**DOI:** 10.3390/s20102967

**Published:** 2020-05-24

**Authors:** Zeming Yang, Cai Li, Zhenzhao Zhang, Guixin Lu, Zifeng Cai, Wenxi Cao

**Affiliations:** 1 State Key Laboratory of Tropical Oceanography, South China Sea Institute of Oceanology, Chinese Academy of Sciences, Guangzhou 510301, China; zmyang@scsio.ac.cn (Z.Y.); zhangzhenzhao@scsio.ac.cn (Z.Z.); luguixin@scsio.ac.cn (G.L.); caizifeng@scsio.ac.cn (Z.C.); wxcao@scsio.ac.cn (W.C.); 2 University of Chinese Academy of Sciences, Beijing 100049, China; 3 Southern Marine Science and Engineering Guangdong Laboratory (Guangzhou), Guangzhou 511458, China

**Keywords:** in situ detection, phosphate, sequential injection analysis, liquid waveguide capillary cell

## Abstract

This study presents an innovative technique for the in situ analysis of aquatic biochemical elements detected through wet chemical processes. A new compact in situ phosphate analyzer based on sequential injection analysis, liquid waveguide capillary flow cell and spectrophotometry was developed, and a safe and modular electronics-chemical separation mechanical structure was designed. The sequential injection system of this analyzer was optimized, and the major functions of this analyzer were studied and estimated. With a 10 cm liquid waveguide capillary flow cell and a 6.3 min time cost of detection, the analyzer reaches a detection limit of 1.4 μg·L^−1^ (≈14.7 nM, [PO_4_^3−^]) and a consumption of 23 μL at most for each reagent. This analyzer was operated in situ and online during two scientific research cruises in the Pearl River Estuary and northern South China Sea. The advantages of this analyzer include its simple versatile manifold, full automation, low chemical consumption and electronics-chemical separate safe structure. Long-term in situ performance of this analyzer will be validated in the future.

## 1. Introduction

With the growing human footprint, the enrichment of nutrient inputs and pollution are significantly altering estuarine and coastal ecosystems [1,2,3,4]. As an important routine macronutrient in natural waters [5], excess phosphate can generate noticeable effects on aquatic environments, such as eutrophication and coral reef decline [4,6,7]. Therefore, the quantification of phosphate in various waters is essential and imperative.

Traditional analysis methods of nutrients in natural waters require the collection and freezing of discrete samples and subsequent analysis in the laboratory, and these methods are high cost in time and chemicals, susceptible to contamination of water samples and limited in long-term detection of marine nutrients [8,9,10]. In situ chemical sensors analyze the samples in their primitive condition, obviate the risk of sample degradation or contamination and remove sample preservation and transportation [11]. In addition, miniaturized autonomous in situ instrumentation provided with excellent performances (such as long-term serial monitoring and fast response time) helps to improve the observing capacities in the constantly changing marine environment [12].

The innovation and development of flow injection analysis (FIA) techniques are pivotal in the transition from laboratory detection to in situ and online detection, and these techniques achieve the automation of sample preprocessing and measurement, the miniaturization of instruments and the low consumption of time, samples and reagents [10,13]. The second generation of flow injection analysis, sequential injection analysis (SIA), which provides a more integrated and controllable system and simple manifold [14], has been widely utilized in laboratorial, online and in situ determination of nutrients in natural waters [9,15,16,17].

The phosphomolybdenum blue (PMB) colorimetric method is the most common and widely used technique for the measurement of dissolved reactive phosphorus (DRP) and is suitable for adaptation to field-deployable devices [18,19]. The principle of the PMB method is that in an acidic medium, DRP reacts with ammonium molybdate to form phosphomolybdenum yellow, which is then reduced to PMB by ascorbic acid, and the absorbance is measured at a wavelength of 882 nm [20].

The liquid waveguide capillary cell (LWCC) constructed with Teflon AF manufactured by DuPont provides amorphous fluoropolymers with a refractive index (1.29–1.31) lower than that of water, achieves the total internal reflection of light and has been developed into a commercial alternative for measurements in liquid media and devices [13,21,22]. The LWCC provides low dispersion of the reaction zone, high sensitivity of sample measurement, little solution consumption and lower limit of detection (LOD) of optical instruments [21,22,23]. In aquatic nutrients analysis field, LWCCs are usually combined with automated flow systems and have been widely utilized in trace-nutrient analysis of phosphate, nitrite, nitrate, silicate and ammonium [24,25,26,27]. The commercially available LWCC module (Patents 1) employing Teflon AF from World Precision Instruments (WPI) is applicable to absorbance measurements and is widely used. However, the WPI LWCC module is mainly suitable for bulky laboratory or online detection systems instead of compact in situ analyzers due to its lack of adaptability, watertight structure and miniaturization, and no commercially available in situ phosphate analyzers have been provided with LWCCs at present.

In this study, a new in situ analyzer based on PMB spectrophotometry and SIA incorporated with an innovative miniature watertight LWCC detection module was developed. This analyzer can quickly and automatically measure DRP concentrations in natural waters and features a simple manifold and low consumption of time and chemicals. The system and structure of this analyzer are versatile for a wide variety of chemical and biotic parameters that need to be detected using wet chemical methods. The versatile LWCC module can be easily modified with different LWCC tubes to meet varied requirements. Laboratory experiments were performed to optimize the key parameters of the SIA system and validate the performance of this analyzer with a 10 cm LWCC module. In situ and online experiments were implemented during two scientific research cruises in the Pearl River Estuary and northern South China Sea and the analyzer reliability was verified.

## 2. Materials and Methods

### 2.1. Apparatus

**Figure 1:**
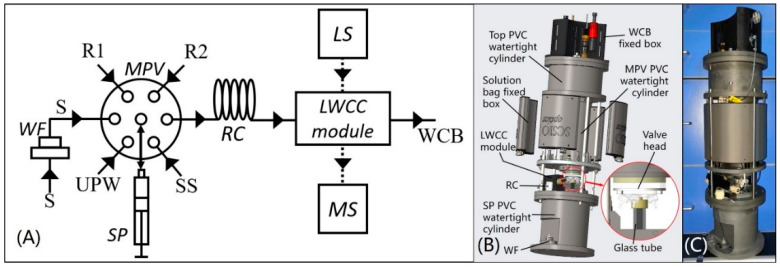
(**A**) System fluidic diagram of the analyzer, where LS = miniature halogen light source, MS = microspectrometer, MPV = multiposition valve, SP = high precision syringe pump, WF = water sample filtration module, RC = reaction coil, S = water sample, UPW = ultrapure water, SS = standard solution, R1 = mixed solution of ammonium molybdate, sulfuric acid and antimony potassium tartrate, R2 = ascorbic acid solution, WCB = waste collection bag; (**B**) three-dimensional model of the analyzer; (**C**) photo of the analyzer.

The analyzer (Patents 2) was based on SIA and spectrophotometry and its SIA fluidic diagram is shown in Figure 1A, the three-dimensional model and photo of the analyzer are, respectively shown in Figure 1B,C. The analyzer is 53 cm height, 17.5 cm in diameter and weighs 8 kg in air and the structure of this analyzer shown in Figure 1B is electronics-chemical separate to protect the mechanical and electronic parts of the analyzer from wet chemicals.

As shown in Figure 1A, the core of the SIA system is the combination of the multiposition valve (MPV) and high precision syringe pump (SP). The MPV and SP were modified from R46S10 (BEIONFLUID, Shanghai, China) and S60 (BEIONFLUID, Shanghai, China) to make them suitable for in situ detection. The combination of the MPV and SP achieves automation of injection and flow in a simple fluidic system. The spectrophotometric system comprises the miniature halogen light source (LS, LS-1, Wyoptics, Shanghai, China), a 650–1100-nm microspectrometer (MS, STS-NIR, OceanOptics, Largo, FL, USA), two waterproof optic fibers (Sun Telecom, Shanghai, China) and an LWCC module.

In Figure 1B, the LS, MS and PCBs for drive control and data acquisition were fixed in the top PVC watertight cylinder and the MPV and SP were sealed in the middle MPV and bottom SP PVC watertight cylinders, respectively, and a quartz glass tube was connected between MPV head and SP. The quartz tube was used for bidirectional mixing of reagents and the sample and the reaction coil (RC) was used for further mixing. RC and the LWCC module were fixed on the cap of the SP PVC watertight cylinder and the water sample filtration module (WF) was fixed outside of the SP watertight PVC cylinder. R1, R2, standard solution (used to calibrate the standard curve) and ultrapure water (UPW) were prepared and injected into medical-grade PVC bags (all solutions and UPW can be used over a month) and these bags were subsequently placed into 3D printing reagent cartridges fixed around the MPV PVC watertight cylinder. All liquid flow tubes and relevant fittings, MPV valve head and quartz glass tube are exposed to work environment in order to prevent the electromechanical components from chemical corrosion.

**Figure 2:**
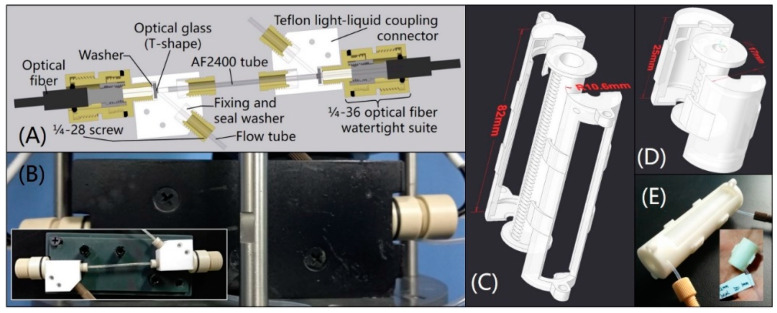
(**A**) Cross-section of the liquid waveguide capillary cell (LWCC) module; (**B**) photo of the LWCC module; (**C**) three-dimensional model of tube looper for internal diameter (E.D.) = 0.8 mm, external diameter (E.D.) = 1.6 mm, length = 1989 mm Teflon tube, total tube volume is 1 mL; (**D**) three-dimensional model of tube looper for I.D. = 1 mm, E.D. = 2 mm, length = 382 mm Teflon tube, total tube volume is 0.3 mL; (**E**) photos of above 1 mL and 0.3 mL tube loopers.

The WF used to obtain analyzable water samples is composed of a 40-mesh copper sieve and a 316 L stainless steel sintered filter. The cross-section of the versatile watertight LWCC module is shown in Figure 2A. Two self-designed PEEK 1/4-36 optical fiber watertight suites were joined to connect and seal the light path of the light-liquid coupling connectors in Figure 2A and one end of the optical fibers used in the analyzer. In this study, an AF2400 tube (Biogeneral, San Diego, CA, USA) at a length of 10 cm, inner diameter (I.D.) of 1 mm and external diameter (E.D.) of 1.6 mm was used in the analyzer to verify the reliability of the in situ LWCC module. The LWCC module was protected by a 3D printing collision-proof cover and the photo is shown in Figure 2B. The RC was made by a Teflon tube (Runze Fluid, Nanjing, China) wound in a patented 3D printing tube looper (Figure 2C–E) (Patents 3). The tube looper was designed to make it convenient for the users to accurately wind reagent tubes, decrease space usage and prevent the tubes from collision, its structure is adaptive to almost all kinds of reagent tubes. 500 kPa hydraulic tests were successfully conducted on this analyzer before in situ tests. The analyzer was operated fully automatically with a small self-contained and watertight 24 V rechargeable lithium-ion battery pack or direct 24-V DC supply.

### 2.2. Reagents

All reagents were analytical pure grade and prepared in ultrapure water (resistivity ≥ 18.2 MΩ·cm^−1^) and were purchased from Guangzhou Chemical Reagent Factory (Guangzhou, China). Ammonium molybdate solution of 0.14 g·mL^−1^ was prepared by dissolving 14 g ammonium molybdate ((NH_4_)_6_Mo_7_O_24_·4H_2_O) in 100 mL ultrapure water and antimony potassium tartrate solution of 0.03 g·mL^−1^ was prepared by dissolving 3 g antimony potassium tartrate (C_4_H_4_KO_7_Sb·1/2H_2_O) in 100 mL ultrapure water. Mixed solution was prepared by adding 45 mL ammonium molybdate solution in 200 mL sulfuric acid solution (*c*(H_2_SO_4_) = 6.0 mol·L^−1^) and then adding 5 mL antimony potassium tartrate solution. Ascorbic acid solution of 0.1 g·mL^−1^ was prepared by dissolving 10 g ascorbic acid (C_6_H_8_O_6_) in 100 mL ultrapure water.

### 2.3. Method

**Figure 3:**
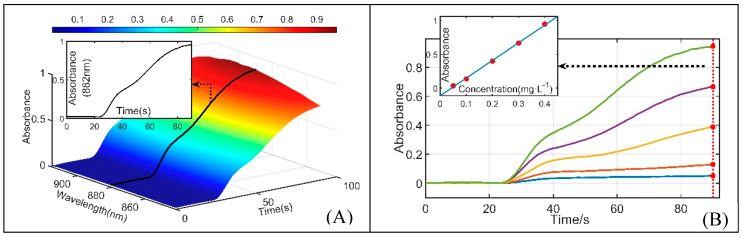
(**A**) Absorbance measurement results over time of incomplete chromogenic reaction; (**B**) absorbance measurement results at characterized band over time and curve fitting of a series of DRP standard solutions at the time of maximum absorbance.

Sufficient reaction time was necessary to achieve complete chromogenic reaction in a standard PMB assay [20]. Incomplete chromogenic reaction was used to reduce detection time in this study to be suitable for in situ measurement. Coupling the MPV and SP, the flow path of the SIA system was cleaned first, and then, the water sample, mixed solution and ascorbic acid solution were sequentially injected into the quartz glass tube in a specific ratio to gradually form an incomplete chromogenic reaction solution after a specific time of reaction (reaction time 1, RT1). The incomplete chromogenic reaction solution was subsequently pushed through the reaction coil and LWCC colorimetric cell at a slow constant speed (corresponding to reaction time 2, RT2), and the absorption spectrum of the flowing reaction solution in the LWCC colorimetric cell was synchronously measured until the SP stopped. During this process, the water sample and reagents were mixed with each other, forming a gradual chromogenic solution. The gradual chromogenic solution can be determined by the absorption spectrum, and the concentration of DRP was ultimately calculated by the Lambert-Beer Law. The synchronous changing process of absorbance is shown in Figure 3A. Figure 3B illustrates the changing process of absorbance at the characterized band over time for a series of DRP standard solutions. Using the curve fitting equation at the position of maximum absorbance, the DRP concentrations of various natural waters can be quickly obtained by this method.

## 3. Results and Discussion

### 3.1. SIA System Optimization

A series of experiments were performed to obtain the most suitable detecting parameters of this SIA system and analyze the effect of sample salinity and carryover. Unless otherwise indicated, all the following optimization experiments were performed with the concentration gradients of 0.05, 0.1, 0.2, 0.3, 0.4 mg·L^−1^ DRP ([PO_4_^3−^]) series standard solutions prepared in ultrapure water.

#### 3.1.1. The Effect of SIA System Parameters

**Figure 4:**
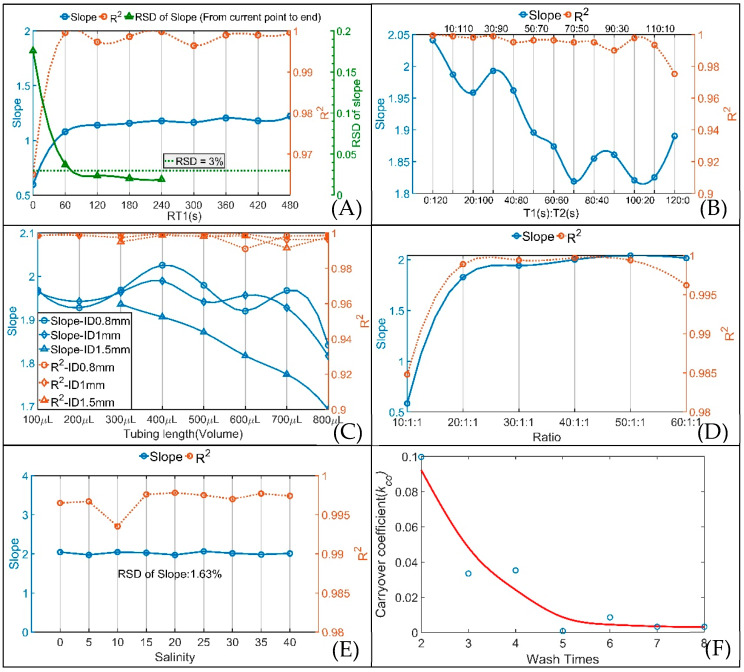
Changes in slope, R^2^ and RSD of the slope for (**A**) different RT1 at a flow rate of 13.3 μL·s^−1^ (corresponding RT2 is 90 s); (**B**) different T1:T2 ratios with RT1 of 120 s at the time of maximum absorbance; (**C**) different reaction coil lengths and I.D.s at the time of maximum absorbance; (**D**) different sample-to-reagent ratios at the time of maximum absorbance; (**E**) different salinities at the time of maximum absorbance; (**F**) change in *k*_CO_ with number of washes.

The influence of different RT1 and RT2 on the SIA system was analyzed. The R^2^ values of the curve fittings for nine RT1 (0, 60, 120, 180, 240, 300, 360, 420 and 480 s) and nine RT2 (20, 30, 40, 50, 60, 70, 80, 90 and 100 s, the corresponding flow rates were 60, 40, 30, 24, 20, 17, 15, 13.3 and 12 μL·s^−1^, respectively) are shown in Figure S1 in Supplementary Materials. As the flow rate decreases, R^2^ increases to greater than 0.99, and the measurement results become better. In consideration of the reliability and time consumption, a 13.3 μL·s^−1^ (corresponding RT2 is 90 s) flow rate was applied in this system based on the quantities of R^2^ being greater than 0.99 and 0.999. The changes in slope and R^2^ of the standard curves at the position of maximum absorbance with different RT1 at a 13.3 μL·s^−1^ flow rate and the RSDs of the slopes are plotted in Figure 4A. The RSD at each slope point was calculated by the slopes from each slope point to the last slope point. After two minutes of RT1, the RSDs of the slopes were less than 3%, and the R^2^ values of every RT1 were all above 0.99, indicating that the slopes were basically constant and that the measurement results were stable after RT1 of two minutes. Considering the time consumption, stability and accuracy, a two-minute (120 s) RT1 was adopted for this system.

For PMB, phosphomolybdenum yellow was first formed by the reaction of the water sample and mixed solution, and then reduced to PMB by ascorbic acid solution. To achieve the optimal PMB reaction result for this system, the above two-minute (120 s) RT1 was divided into two waiting times: the waiting time after injecting the water sample and mixed solution (T1) and the waiting time after injecting ascorbic acid solution (T2). The analysis results for different T1:T2 ratios are shown in Figure S2 in Supplementary Materials and Figure 4B. It is obvious from Figure S2 that the measurement result is most stable when T1 (s):T2 (s) = 0:120. As shown in Figure 4B, with an increase in T1 (s):T2 (s), R^2^ and the slopes of the curve fitting results decrease. As a result, T1 (s):T2 (s) = 0:120, that is, proceeding without a pause after injecting the mixed solution, is the optimal choice for this system.

Different reaction coil lengths and I.D.s also cause different mixing effects when the mixing solution is flowing through the reaction coil. In SIA, 0.8 mm and 1.5 mm I.D. tubes give improved precision compared with 0.5 mm I.D. tubes [28]. Considering that the LWCC I.D. is 1 mm, three I.D.s (0.8, 1 and 1.5 mm) and eight reaction coil lengths (corresponding volumes from 100 to 800 μL) for each I.D. were analyzed and the analysis results are plotted in Figure S3 in Supplementary Materials and Figure 4C. By analyzing the slopes of all curve fittings, the slopes for the 0.8 mm I.D. were larger overall than the other slopes, and the maximum slope for the 0.8 mm I.D. occurs at 400 μL. Therefore, the combination of the 796 mm tube length (corresponding volume is 400 μL) and 0.8 mm tube I.D. was optimal for the SIA system.

To reduce reagent consumption and lower LOD, experiments with sample-to-reagent ratios from 10:1:1 to 60:1:1 were performed to analyze the effect of the sample-to-reagent ratio in the SIA system, and the results are shown in Figure S4 in Supplementary Materials and Figure 4D. The 50:1:1 ratio was chosen to be applied in this analyzer because of the relatively large slope and reasonable R^2^ for this ratio.

#### 3.1.2. The Effect of Sample Salinity

Studying the effect of salinity was necessary for the potential application of this analyzer in various natural waters. DRP ([PO_4_^3−^]) series standard solutions of 0.05, 0.1, 0.2, 0.3 and 0.4 mg·L^−1^ prepared in artificial seawater at 9 salinities (ranging from 0 to 40 with an interval of 5) were used to study the effect of salinity on the analyzer, and the results are shown in Figure 4E and Figure S5 in Supplementary Materials. All the R^2^ values obtained from curve fitting were above 0.99, and the RSD of the slopes for different salinities is 1.63%; these results indicate that the effect of salinity on the analyzer was negligible and that a salinity correction was not required in the analyzer.

#### 3.1.3. Carryover Effect

Carryover is the degree to which the result obtained is influenced by the concentration of the preceding sample [29], it is an important parameter in monitoring system performance [30]. The carryover coefficient, *k_CO_*, was used to quantify the carryover effect and is calculated by the following equation [30,31].

$$k_{co}=\left(A_{i}-A_{i-2}\right)/A_{i-1}$$

where *k*_CO_ is the carryover coefficient and *A_i−_*_2_, *A_i−_*_1_ and *A_i_* are the measured absorbances of samples *i* − 2 (low concentration), *i* − 1 (high concentration) and *i* (low concentration), respectively.

The number of times the SIA system is washed directly determines the carryover effect of the analyzer. A 0.5 mg·L^−1^ DRP standard solution was used as the high-concentration sample and a blank was used as the low-concentration sample to give more reliable measurements of *k_CO_* and the results are plotted in Figure 4F. After 5 washes, *k_CO_* was basically stable at 0.003. By analyzing the corrected absorbance and *k_CO_*, the 0.5 mg·L^−1^ DRP standard solution had an effect on the following blank sample of 0.0012 mg·L^−1^, which is lower than the LOD of the analyzer, indicating the negligible impact of carryover on the analyzer. Considering time consumption, 5 washes was executed in the SIA system.

### 3.2. Performances

By analyzing the standard solutions and natural water samples, the recovery, LOD and linear range of the analyzer were obtained to verify its performance.

The blank recovery and sample recovery of various water samples were analyzed, and the results are listed in Table 1. The recoveries ranged from 93.3% to 106.8%—and combined with the above study on salinity—the results indicate that this analyzer is suitable for most natural waters. The LOD (calculation method is shown in SI) and linear range of the analyzer with 10 cm LWCC and 6.3 min analysis time are 1.4 μg·L^−1^ (≈14.7 nM, [PO_4_^3−^]) and 0.0046–0.8 mg·L^−1^.

A complete measurement by this analyzer takes a total of 6.3 min and involves washing (5 times), sample injection, reaction and detection. Compared with the measurement times of commercially available DRP analyzers, such as 15 min for the HydroCycle-P (Sea-Bird Coastal, Bellevue, WA, USA), 30 min (for a full four-parameter cycle) for the Wiz Probe (Systea S.p.A. Analytical Technologies, Anagni, Italy) and 12 min for the EcoLAB II (Green Eyes LLC, Easton, MD, USA), the analyzer in this study greatly shortens the in situ measurement time of DRP.

### 3.3. Method Comparison

Seawater at depths of 5–10 m was collected from the Pearl River Estuary and northern South China Sea by an SBE 32 Carousel Water Sampler and river water from the Pearl River was also collected. The collected seawater and river water samples were analyzed in the laboratory by a LAMBDA 650S UV-Vis Spectrophotometer (PerkinElmer, Waltham, MA, USA) and the DRP analyzer of this study to validate the analyzer reliability. A comparison of the analyzer and spectrophotometer detection results is shown in Figure S6 in Supplementary Materials, which shows the good agreement (R^2^ = 99.7%, slope is 0.927) between the two methods and the dependability of this analyzer.

### 3.4. In situ and Online Measurements and Data Analysis

**Figure 5:**
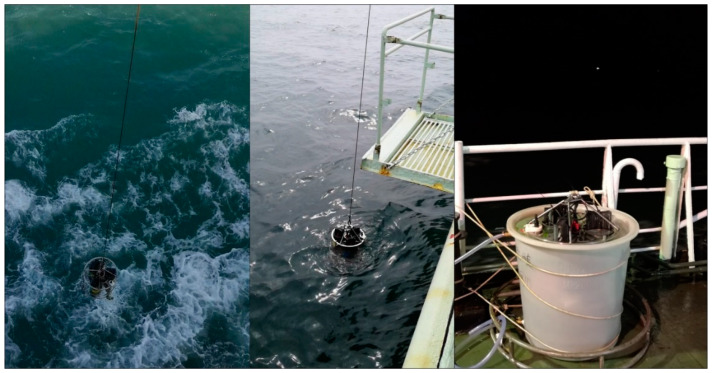
Photos of the analyzer being pulled out of seawater during in situ experiments on SHIYAN 1 (**left**) and SHIYAN 2 (**middle**); photo of online experiment (**right**).

A series of in situ and online experiments were performed to verify the detection performance of the analyzer during the “Healthy Ocean” scientific research cruise on the R/V *SHI YAN 2* from 25 October 2019 to 1 November 2019 and the “Instrument Acceptance” scientific research cruise on the R/V *SHI YAN 1* from 29 November 2019 to 5 December 2019, in the Pearl River Estuary and northern South China Sea. An SBE 37-SM MicroCAT (S/N: 37-10819) was paired with this DRP analyzer to record the CTD data during both cruises, and online experiments were carried out by placing the analyzer in a water tank on the deck because of the fierce sea conditions. Figure S7 in Supplementary Materials provides maps of the two cruise locations and photos of in situ and online experiments are illustrated in Figure 5.

**Figure 6:**
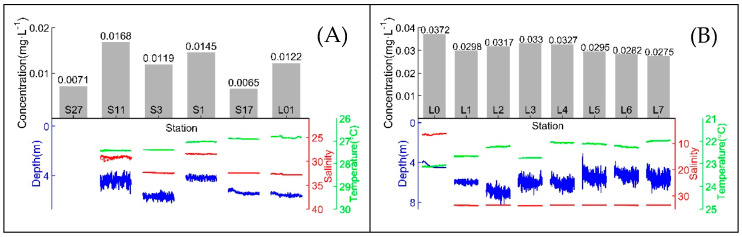
(**A**) In situ measurement results of the “Healthy Ocean” cruise and corresponding CTD data during underwater experiments; (**B**) in situ measurement results of the “Instrument Acceptance” cruise and corresponding CTD data during underwater experiments.

The measurement results of all in situ stations and corresponding CTD data during underwater experiments on the “Healthy Ocean” and “Instrument Acceptance” cruises are shown in Figure 6 and Table 2 lists the online measurement results and CTD data of the above two cruises.

The detection results obtained on the “Healthy Ocean” cruise indicate that the distribution of DRP concentrations in the Pearl River Estuary and northern South China Sea was between 0.005 and 0.025 mg·L^−1^ in October 2019 and this result agrees with research by Jiang et al. in October 2015 [32]. The Pearl River Estuary in situ DRP concentration detection results obtained on the “Instrument Acceptance” cruise are consistent with water sample analysis results by Liu et al. at a similar location in October 2015 [33] and are also consistent with research by Jiang et al. in October 2015 [32]. The reliability of field detection by this DRP analyzer was verified.

## 4. Conclusions

This study proposes an SIA-based DRP in situ spectrophotometric analyzer with an integrated versatile LWCC colorimetric cell. A series of experiments were conducted to optimize the SIA system and the technical feasibility and method reliability of this in situ analyzer was verified by field measurements and laboratory detection. This analyzer also provides a solution for in situ analysis of biochemical elements that need to be analyzed by a wet chemical method. Further studies are required on more high-pressure-resistant components and structures as well as more miniature and integrated valves and pumps to make the in situ analyzer more compact and provide it with deep sea detection capability for profile detection. In addition, studies on chemical detection are necessary to decouple chemical and biotic effects on phosphate in aquatic environments, and research on LWCC module with LWCC length of 1 m or more will carry out to enhance sensitivity and decrease the LOD to achieve oligotrophic ocean detection.

## 5. Patents

1. Liu, S.Y. Detecting analyte in solvent stream for micro-chemical analysis utilising flow through detectors-using light absorption and fluorescence as measure of chemical properties of small amounts of flowing fluid analyte, esp. in conjunction with liq. chromatography and capillary electrophoresis. US5444807-A, 1995-08-22.
2. Li, C.; Yang, Z.; Xu, C.; Zhang, Z.; Gou, M.; Lu, G.; Cao, W.; Yang, Y. An in situ analysis device for the detection of nutrients in seawater. ZL201810904083.5, 2019-10-08.
3. Li, C.; Yang, Z.; Gou, M.; Xu, C.; Cao, W. A multi-adaptive tube looper for wet chemical measurement. ZL201710764520.3, 2019-10-15.

## Figures and Tables

**Table 1:** Blank recovery and sample recovery of various water samples.

Sample	Salinity	Concentration (mg·L^−1^)	Added (mg·L^−1^)	Found (mg·L^−1^)	Recovery (%)
Standard solution (prepared with pure water)	0	0.05	/	0.0532	106.4
		0.15		0.1463	97.5
		0.25		0.2481	99.2
		0.35		0.3547	101.3
		0.4		0.4036	100.9
Standard solution (prepared with artificial seawater)	32	0.05	/	0.0482	96.4
		0.1		0.0933	93.3
		0.2		0.1961	98.1
		0.3		0.2933	97.8
		0.4		0.4014	100.3
River water (Pearl River)	0	0.1464	0.1	0.2428	96.4
			0.2	0.3491	101.3
			0.3	0.4593	104.3
Seawater 1	33.7	0.0534	0.025	0.0771	94.8
			0.05	0.1068	106.8
			0.1	0.1529	99.5
Seawater 2	33.5	0.0566	0.025	0.0805	95.6
			0.05	0.1088	104.4
			0.1	0.1584	101.8
Seawater 3	33.6	0.0547	0.025	0.0795	99.2
			0.05	0.1063	103.2
			0.1	0.1554	100.7

**Table 2:** Online measurement results of the “Healthy Ocean” and “Instrument Acceptance” cruises.

Cruise	Station	DRP Concentration (mg·L^−1^)	Salinity	Temperature (°C)
Healthy Ocean	S0	0.0117	2.0	26.5
	S12	0.0247	30.33	25.8
	S13	0.0056	30.29	25.82
	S14	0.0228	28.75	25.15
	S16	0.0172	27.53	24.92
	S18	0.0196	30.37	25.88
	S25	0.0118	30.2	25.6
	S31	0.0217	31.4	26.4
Instrument Acceptance	L0	0.0278	7.28	24.18

Note: In situ and online tests were both performed at the L0 station of the “Instrument Acceptance” scientific research cruise.

## Supplementary Materials

Supplementary Materials associated with this article are available online at https://www.mdpi.com/1424-8220/20/10/2967/s1, Figure S1: R^2^ values of curve fittings for different reaction times and push speeds, Figure S2: R^2^ values of curve fittings for different T1:T2 ratios and a total reaction time of 120 s, Figure S3: R^2^ values of curve fittings for different reaction coil lengths and IDs, Figure S4: R^2^ values of curve fittings for different sample-to-reagent ratios, Figure S5: R^2^ values of curve fittings for different salinities, Figure S6: Comparison of the analyzer and spectrophotometer detection results, Figure S7: Locations of the stations on the two cruises. (**A**): “Healthy Ocean” cruise; (**B**) “Instrument Acceptance” cruise.
