# Supplementary material for: Development of an In Situ Analyzer Based on Sequential Injection Analysis and Liquid Waveguide Capillary Flow Cell for the Determination of Dissolved Reactive Phosphorus in Natural Waters
Source: Sensors (Basel). 2020 May 24;20(10):2967. doi: 10.3390/s20102967 (PMC7288038; doi:10.3390/s20102967)
Supplement: Supplementary file 1 [file sensors-20-02967-s001.pdf]

Supplementary Information

Development of an In Situ Analyzer Based on Sequential Injection Analysis and Liquid Waveguide Capillary Flow Cell for the Determination of Dissolved Reactive Phosphorus in Natural Waters

Zeming Yang [1,2], Cai Li [1,3,*], Zhenzhao Zhang [1], Guixin Lu [1], Zifeng Cai [1] and Wenxi Cao [1]

[1] State Key Laboratory of Tropical Oceanography, South China Sea Institute of Oceanology, Chinese Academy of Sciences, Guangzhou 510301, China; zmyang@scsio.ac.cn (Z.Y.); zhangzhenzhao@scsio.ac.cn (Z.Z.); 11363996@qq.com (G.L.); caizifeng@scsio.ac.cn (Z.C.); wxcao@scsio.ac.cn (W.C.)

[2] University of Chinese Academy of Sciences, Beijing 100049, China

[3] Southern Marine Science and Engineering Guangdong Laboratory (Guangzhou), Guangzhou 511458, China

* Correspondence: liclaire@scsio.ac.cn

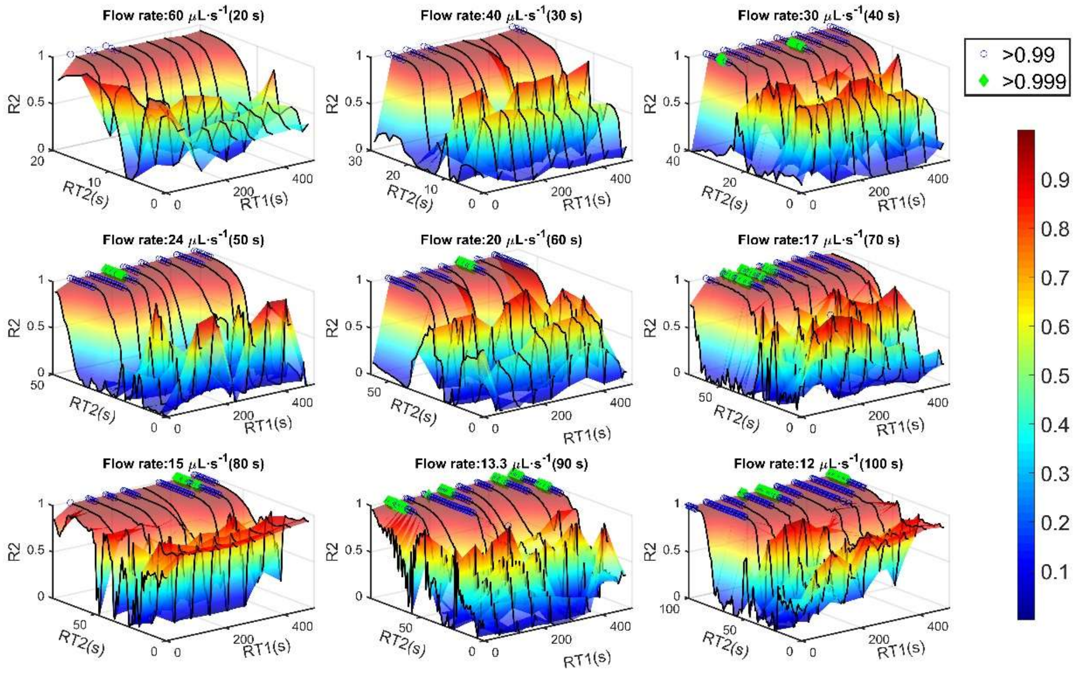

Figure S1. R^2 values of curve fittings for different reaction times and push speeds.

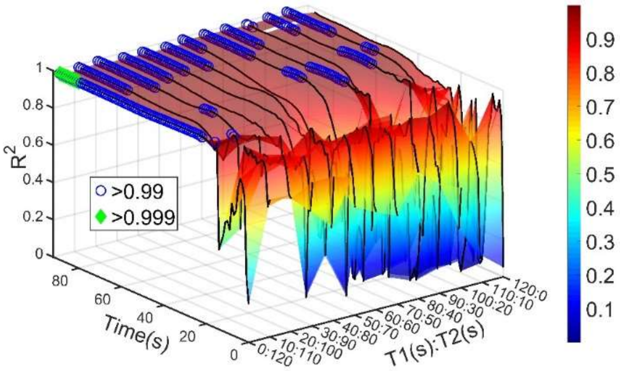

Figure S2. R^2 values of curve fittings for different T1:T2 ratios and a total reaction time of 120 s.

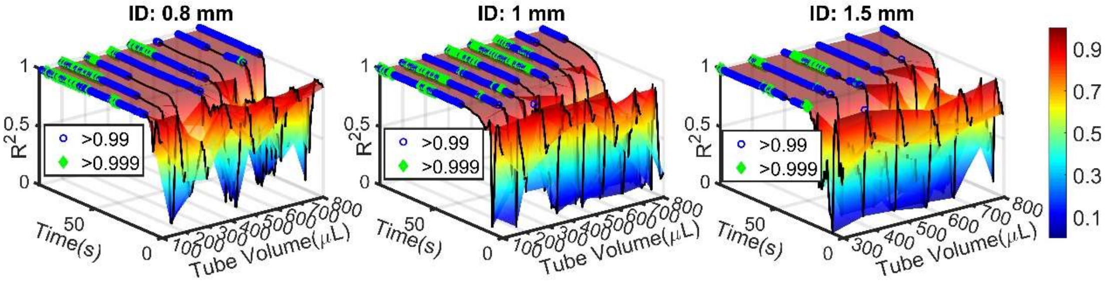

Figure S3. R^2 values of curve fittings for different reaction coil lengths and IDs.

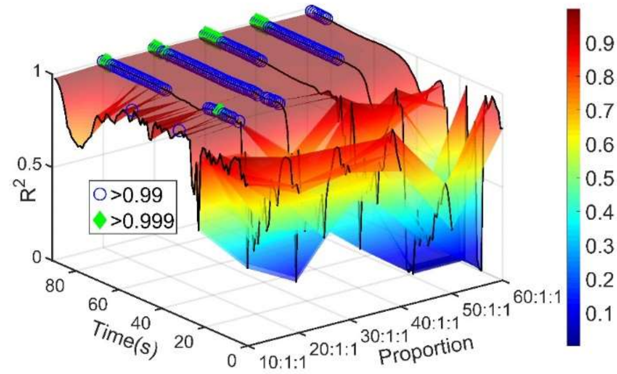

Figure S4. R^2 values of curve fittings for different sample-to-reagent ratios.

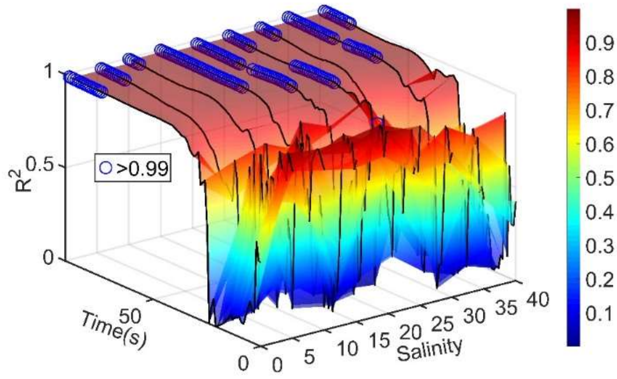

Figure S5. R^2 values of curve fittings for different salinities.

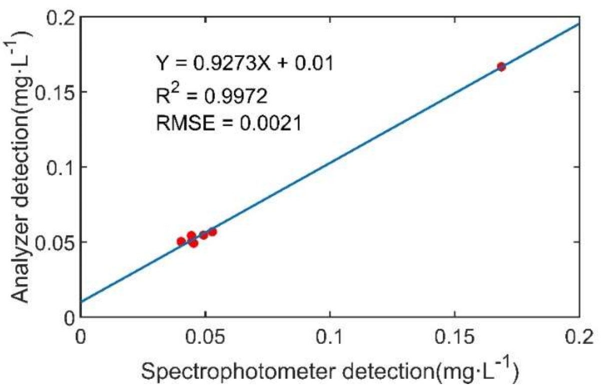

Figure S6. Comparison of the analyzer and spectrophotometer detection results.

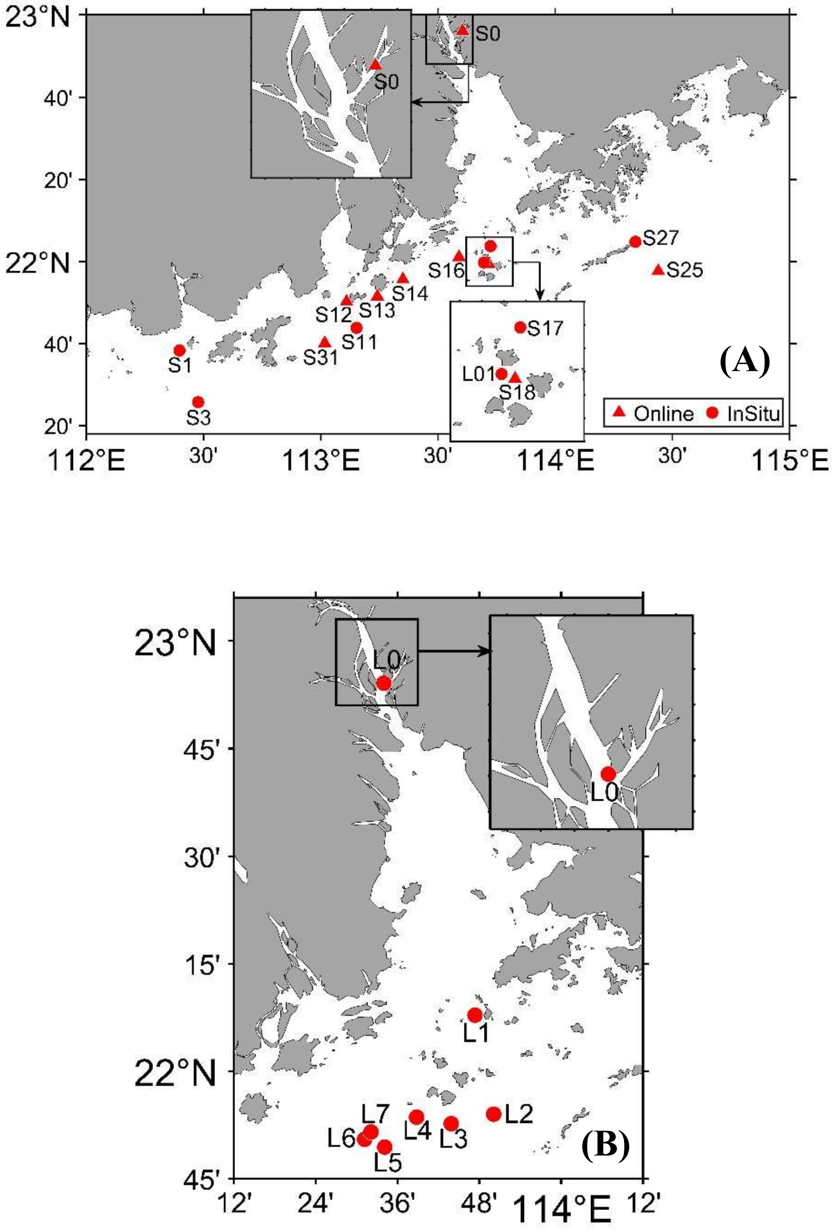

Figure S7. Locations of the stations on the two cruises. (A): "Healthy Ocean" cruise; (B) "Instrument Acceptance" cruise.

LOD calculation method:

The LOD is calculated by the following equation:

$$\mathrm{LOD} = \frac{3S_0}{k}$$

Where S_0 is the standard deviation of the blank samples for multiple parallel determinations, and k is the slope of the standard curve.

With this analyzer, the S_0 (n = 10) is 0.0011 and k is 2.39 (the concentration unit is mg·L^{-1} of the standard curve), so the LOD is about 1.4 μg·L^{-1} and its corresponding molar concentration is 14.7 nM (the molecular weight of PO_4^{3-} is 95).
